# Moderated Mediation Mechanism to Determine the Effect of Gender Heterogeneity on Green Purchasing Intention: From the Perspective of Residents’ Values

**DOI:** 10.3389/fpsyg.2021.803710

**Published:** 2022-01-25

**Authors:** Xianchuan Yang, Santhaya Kittikowit, Tim Noparumpa, Jiayun Jiang, Shih-Chih Chen

**Affiliations:** ^1^School of Business, Wuxi Vocational Institute of Commerce, Wuxi, China; ^2^Synergetic Innovation Research Base of Digital Business Development of Jiangsu Province, Wuxi, China; ^3^Project Team of Bellwether Talents Scheme, Wuxi Vocational Institute of Commerce, Wuxi, China; ^4^Chulalongkorn Business School, Chulalongkorn University, Bangkok, Thailand; ^5^Department of Information Management, National Kaohsiung University of Science and Technology, Kaohsiung, Taiwan

**Keywords:** gender heterogeneity, altruistic values, egoistic values, green purchasing intention, media exposure

## Abstract

This study determines gender differences in the generation logic for green purchasing intention within the framework of bounded morality and bounded self-interest and determines the causes of the attitude–behavior gap from a new perspective. Empirical analysis of 977 sample data points is used to test the influencing mechanism of gender heterogeneity on green purchasing intention through altruistic values (ALVs) and egoistic values (EGVs). Meanwhile, the moderated mediation effects are also analyzed. The results show that gender heterogeneity negatively affects ALVs and positively affects EGVs for women as the reference group. The mediation effect of ALVs and EGVs is significant, and there are significant gender differences in the formation of values and green purchasing intention. As expected, women demonstrate higher levels of proenvironmental intention than men. Media exposure (ME) significantly moderates the mediation models. It negatively moderates the mediation effect of ALVs and positively moderates the mediation effect of EGVs. The results reveal the complex formation mechanism for green purchasing intention. It can conclude that the gender differences in terms of green purchasing, the different guiding roles of dual values, and the moderated mechanism of ME are key elements in accurate guidance of green consumption and the effective modification of the attitude–behavior gap.

## Introduction

Building an ecological civilization is a global development strategy for the future. This strategic concept prevents environmental pollution and ecosystem degradation and is an inherent requirement for achieving sustainable development (Costa et al., 2021; Rasool et al., 2021). The cultivation of an ecological civilization requires the leadership of ecological thought and the exploration of scientific realization paths. Of the various factors that trigger environmental crises, non-green consumption is considered as one of the main causes and studies show that 40% of environmental problems are directly attributable to non-green consumption. Furthermore, the environmental pollution on the production side is mostly demand-driven (Yang and Zhang, 2021). Therefore, governments and environmental organizations at all levels have taken measures to actively persuade manufacturers and consumers to become greener (Uddin et al., 2021). There have been successes but green consumption is not stable and is not a conscientious and initiative: it is sometimes seen as token and formalistic environmental behavior (Dangelico et al., 2021; Szabo and Webster, 2021). Attitude–behavior gap is widespread (Peattie, 2010), so a steady and effective path to a greener society is necessary (Yadav and Pathak, 2017; Mi et al., 2020).

Green consumption has been the subject of many studies, which construct a more explanatory integrated or extended model for green consumption based on a mature theoretical framework from various perspectives, such as ecological personality (Yang and Zhang, 2021), motivation (Gilal et al., 2020), social norms (Kim and Seock, 2019; Akhtar et al., 2021), self-identity (Lee, 2009; Khare and Pandey, 2017; Neves and Oliveira, 2021), mindfulness (Barbaro and Pickett, 2016), and consumption value (Woo and Kim, 2019). These studies have promoted the expansion of green consumption and methods to overcome the attitude–behavior gap among residents but further study is required. Some studies overemphasize the altruistic (egoistic) appeals of green consumption (Han, 2015) and ignore the reasonable egoistic (altruistic) appeals of individuals, which leads to extreme environmental altruism (egoism) (Yang et al., 2020). Some studies use a research framework that considers dual appeals to address this research gap, but this strategy does not fully detail the generation process of dual appeals itself and the complex influence mechanism for green consumption (Le et al., 2019). Some studies determine the effect of gender heterogeneity on green consumption (Mostafa, 2007; Lee, 2009; Pinto et al., 2014), but fundamental mechanisms are not determined. In particular, the specific influence path of gender heterogeneity on green consumption has not been clearly mapped, and few studies consider the attitude–behavior gap from the perspective of gender differences. Other studies determine the role of contextual factors such as media in achieving green consumption and note that psychological mechanisms have a key influencing power on behavioral decisions (Luo et al., 2020). These studies advocate to explore the formation mechanism for green consumption and the causes of the attitude–behavior gap from the potential moderating role of contextual factors. However, empirical studies on this issue are not yet sufficient.

Innovation diffusion theory (Rogers, 1983) states that media communication is more advantageous in the breadth of information diffusion and is an important external source of information processing for individuals of different genders, which may have a potential moderating effect on the process of generating green purchasing intention. However, there is no consensus on the effectiveness of the information repetition strategy (e.g., Sawyer, 1973; Cacioppo and Petty, 1979). Specifically, Eisend (2006) failed to identify the exact repeat effect by meta-analysis. Some studies determine the influence of media exposure (ME) as a predictor (Ivanova et al., 2019; Yang et al., 2020), but no attempt has been made to determine its effect as a moderator.

Values have orientation, recognition, and motivation functions for individual daily behavior (Stern et al., 1999; Stern, 2000; Lan et al., 2009; Han, 2015) and guide and regulate the generation and choice of behaviors (Uddin et al., 2021). Values are also stable so they can better predict and explain the generation of behavioral intention (Xu et al., 2021). In the original model and the improved model of the value-belief-norm (VBN) theory, altruistic values (ALVs) and egoistic values (EGVs) reflect well an individual’s dual appeals. Therefore, this study uses ALVs and EGVs that reflect dual appeals from VBN theory as the predictor variables for green purchasing intention (Stern, 2000; Kiatkawsin and Han, 2017) and determines the mechanism by which gender heterogeneity influences green purchasing intention through altruistic and egoistic appeals within the framework of bounded morality and bounded self-interest.

Moreover, recent studies have explored the mediated mechanism or moderated mechanism in the research framework of proenvironmental behavior (Chen and Chang, 2013; Pinto et al., 2014; Ivanova et al., 2019; Gilal et al., 2020). Nevertheless, most studies neglect the moderated mediation effect. The integration of these two processes is more in line with the reality of green consumption, and a moderated mediation framework may increase understanding of how green purchase activities are generated (Yang et al., 2020). Thus, this study constructs a moderated mediation model to theoretically show how marketers and policymakers can increase green purchases.

To address the research gaps, this study has the following goals: It seeks to verify the general generation logic of individuals’ green purchasing intention in terms of the influence of gender heterogeneity and compares the two mediated paths of ALVs and EGVs. It seeks to test the potential moderating effect of frequency of ME in terms of environmental information (abbreviated as ME) on the research model and to generate empirical evidence for optimizing precise persuasion strategies in the media. It seeks to decompose the values connotation and to determine the specific causes of the attitude–behavior gap from a new perspective, such as gender difference and the causal chain of attitude–intention–behavior, to increase the efficiency of environmental governance in the green market.

## Theoretical Background and Hypothesis Development

According to VBN theory, as proposed by Stern et al. (1999), Stern (2000) proenvironmental behavior is driven by individual values, and proenvironmental intention or behavior is the result of the joint influence of ALVs and EGVs (Le et al., 2019; Yang and Zhang, 2021). Individuals with ALVs focus on human interests and care about the long-term interests of others and society (Xu et al., 2021). Individuals with EGVs are more concerned with their own interests and self-improvement and avoid possible harm to themselves due to changes in the external environment (Kiatkawsin and Han, 2017) and attempt to influence and control others to achieve their personal goals.

Purchasing intention is a psychological construct that includes subjectivity (Fishbein and Ajzen, 1975) and reflects the probability that individuals perform a specific behavior (Woo and Kim, 2019). Fishbein and Ajzen (1975) pioneered the concept of purchasing intention in the field of consumer’s behavior. Their theory of reasoned action posits that behavioral intention acts as a mediator of the relationship among attitudes, subjective norms, and behavior. Therefore, green purchasing intention refers to the subjective propensity of consumers to purchase green products and the probability of implementation (Yadav and Pathak, 2017), which is often used as a substitution variable for behavior to predict green purchasing behavior because actual sampling data for purchasing behavior are difficult to measure.

### Mediation Effect of Dual Values

Biological differences and heterogeneous social experiences contribute to individual gender heterogeneity (Pinto et al., 2014), so gender heterogeneity has a significant effect on individual thought patterns and behavioral styles (Mostafa, 2007). Fukukawa et al. (2007) noted that women are more concerned with social justice, harmony with nature and environmental protection; men are more concerned with personal success, competence development, and life ambitions (Lan et al., 2009). Therefore, gender heterogeneity has a different impact on pursuing individual life goals, in that women tend to be social goal-oriented, focus on interpersonal harmony (relationship-oriented), and exhibit more friendly, collectivistic, and selfless behavior (Meyers-Levy, 1988). Men are self-goal-centered, task-oriented, and behave in ways that are characterized by harshness, competition, and control over others (Pinto et al., 2014).

Gender heterogeneity can have a significant effect on shaping power of individual values, and different values (ALVs and EGVs) produce different degrees of environmental concern (Lee, 2009; Le et al., 2019). These differences in environmental concern are formed during early education and generate a different sense of environmental responsibility (Yang and Zhang, 2021). The effect of gender heterogeneity on dual values is represented by occupation of materials, and Pinto et al. (2014) demonstrated that gender heterogeneity significantly affects sustainable consumption. Women are more willing than men to change lifestyle and use dual values to reduce negative environmental effect. They are more sensitive to environmental issues (Lee, 2009) and are willing to use more green products to reduce pressure on the environment from consumption for the next generation. Accordingly, the following hypotheses are defined.

*H1: Gender heterogeneity indirectly influences residents’ green purchasing intention through the joint effects of ALVs and EGVs.*
*H2: Gender heterogeneity indirectly affects residents’ green purchasing intention through ALVs.*
*H3: Gender heterogeneity indirectly affects urban residents’ green purchasing intention through EGVs.*

### Moderated Mediation Effects of Media Exposure

Media exposure refers to the frequency of exposure to environmental crisis information that is disseminated by the media (Ivanova et al., 2019; Yang and Zhang, 2021). This is a persuasive strategy that is regularly practiced by the media. Some studies describe this as media persuasion frequency (Yang et al., 2020), whereby environmental advocates and green marketers spread various environmental crisis information at high frequency on multiple media platforms to change audiences’ attitudes and behaviors (Yang and Zhang, 2021).

Different gender subgroups have significantly different cognitive styles, which is a major cause of individual disparity (Meyers-Levy, 1988). Beynon et al. (2010) stated that women show more patience and delicacy than men in terms of the receipt and processing of information, and the same difference also affects comparison decision-making (Meyers-Levy and Sternthal, 1991). Meyers-Levy (1988) argued that men are task-oriented and tend to be goal association oriented in their screening of external information, but women, as a socially oriented group, are more susceptible to messages with emotional and humane components. Barber and Terrance (2001) noted that men are calm and sometimes overconfident in terms of risk-taking, but female consumers are cautious and more patient, especially in terms of green consumption, when faced with high-frequency exposure to media reports of an environmental crisis. Women show a higher environmental sensitivity than men (Lee, 2009), they relate more to nature (Yang et al., 2020), and ecological neuroticism causes women to be more prone to mood swings in response to the environmental problems that are described in the media. Ecological agreeableness and ecological conscientiousness mean that women are more concerned to ensure a harmonious coexistence between man and nature (Yang and Zhang, 2021). High-frequency exposure to media information about environmental crises information tends to awaken female’s social aspirations, and they focus more on the health and life quality of family members and friends than men (Pinto et al., 2014). Women are more efficient in shaping ALVs so they consciously resist non-environmental behaviors and are willing to use green products. However, EGVs produce the opposite effect.

As lower frequency exposure to environmental crisis information in the media, positive environmental emotions such as environmental literacy, empathy, and helpfulness are suppressed among women (Lan et al., 2009), and altruistic guidance is not activated but male awareness of self-improvement is fully released, and the pursuit of personal success and demonstration of competence prevails over environmental concerns, so personal norms are not generated (Yang and Zhang, 2021). The opposite is true for EGVs. Lower frequency exposure to media reports is not conducive to building environmental social norms and can prevent the shaping of ALVs and accelerate the generation of EGVs. A lack of group pressure means that men are more likely to engage in overconsumption and non-green consumption (Pinto et al., 2014). Taken together, the following hypothesis is proposed.

*H4.* ME *significantly moderates the mediation effect of ALVs between gender heterogeneity and residents’ green purchasing intention. Using women as the reference group, the greater the ME, the weaker is the mediation effect of ALVs, and vice versa.*
*H5. ME significantly moderates the mediation effect of EGVs between gender heterogeneity and residents’ green purchasing intention. Using women as the reference group, the greater the ME, the greater is the mediation effect of EGVs, and vice versa.*

**FIGURE 1:**
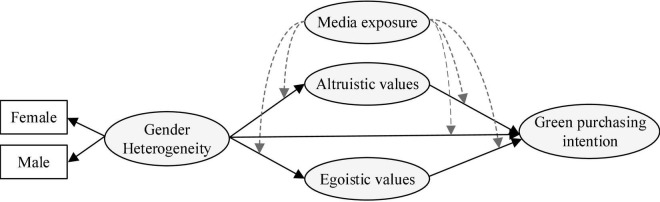
Research model.

Using these hypotheses, this study determines the mechanisms by which gender heterogeneity affects green purchasing intention through ALVs and EGVs and explores the potential moderating effect of social contextual factors (ME) on the mediation model. The research model is shown in Figure 1.

## Methodology

### Data Collection and Sample Characteristics

The respondents were chosen from the Yangtze River Delta City Group, which is the most economically developed region in China and is one of the six largest city groups in the world. Urban residents of the Yangtze River Delta have relatively sound environmental concepts and knowledge, so a questionnaire survey is possible and the sample is more representative.

**FIGURE 2:**
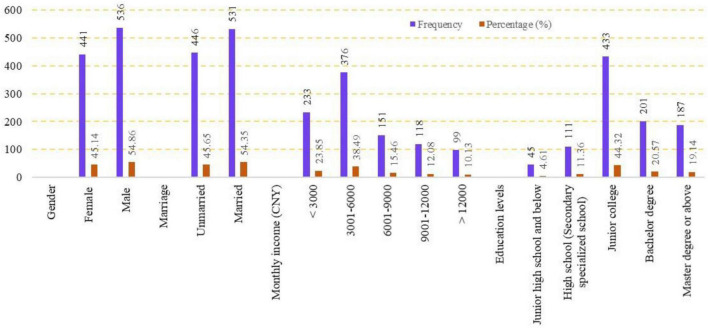
Sample demographic (*N* = 977).

Because of restriction due to the COVID-19 pandemic, this survey was mainly conducted online, on the “www.wenjuan.com” platform. After contacting the potential respondents and explaining the purpose of the questionnaire, electronic questionnaire links were sent *via* QQ and WeChat, and the respondents were instructed to send the links to their work groups, to protect other respondents’ identity. Paper questionnaires are used for random surveys in casual encounters. The survey was completed in September 2020 and lasted about 3 months. A total of 997 valid questionnaires were collected. The screening criteria for valid questionnaires were that samples had a green cognitive bias and social desirability bias and no variation in answers. Outliers are identified using the Mahalanobis distance statistics (Curran et al., 1996). Finally, 977 samples were used for an empirical analysis. The sample profile is shown in Figure 2.

### Measurement

Gender heterogeneity is a binary variable, for which women = 0 and men = 1, with women as the reference group. As shown in Table 1, ALVs (Cronbach’s alpha = 0.842) and EGVs (Cronbach’s alpha = 0.755) are adopted from the study by Kiatkawsin and Han (2017). ALVs contain four items and EGVs contain three items. Green purchasing intention uses the criteria of the study by Yadav and Pathak (2017) and contains three items (Cronbach’s alpha = 0.863). Yang and Zhang’s (2021) four-item scale is used to measure ME (Cronbach’s alpha = 0.879). All scales are derived from a pro-environmental context and are measured using 7-point Likert scale: 1 = strongly disagree or not at all; 7 = strongly agree or always.

**TABLE 1:** Items and original literature (*N* = 977).

Variable	Items	Standardized loadings	Cronbach’s alpha	Composite reliability	References
ALV	Equality, equal opportunity for all.	0.649	0.842	0.854	Kiatkawsin and Han, 2017
	A world at peace, free of war, and conflict.	0.666			
	Social justice, care for the weak.	0.898			
	Helpful, helping others.	0.849			
EGV	Social power, control over others, and dominance.	0.673	0.755	0.763	Kiatkawsin and Han, 2017
	Wealth, material possessions, and money.	0.670			
	Authority, the right to lead or command.	0.810			
GPI	I will purchase green products for personal use.	0.795	0.863	0.869	Yadav and Pathak, 2017
	I am willing to purchase green products for personal use.	0.879			
	I will make an effort to purchase green products.	0.814			
ME	How often do you come across environmental crisis information on television?	0.813	0.879	0.882	Yang and Zhang, 2021
	How often do you come across environmental crisis information in advertisements?	0.874			
	How often do you come across environmental crisis information on radio?	0.769			
	How often do you come across environmental crisis information on the internet?	0.770			

All scales are derived from the green consumption context so well suited to the subject of this study. Translation–back translation is used to translate the English scales, and then, three Ph.D. students whose research interests include consumer’s behavior reviewed the items to ensure the accuracy and validity of the translated scales. To counter any potential negative influence of social desirability bias, one screening question was included at the end of the questionnaire for the pilot and the formal survey, asking respondents to enter the name of a green product that they had purchased in the past year. Samples for which there is an implicit green cognitive bias and social desirability bias are removed (Yang and Zhang, 2021). When the initial scale was created, a pretest was conducted in April 2020 on www.wenjuan.com, and 221 valid samples were obtained. The fourth item in the initial scale of EGVs was then deleted after exploratory factor analysis and reliability analysis.

## Data Analysis and Results

### Descriptive Statistics

The mean, standard deviation (SD), and Pearson’s correlation coefficient for the constructs are listed in Table 2. Correlation analysis shows that gender is negatively correlated with ALVs (*r* = –0.093, *p* < 0.01), positively correlated with EGVs (*r* = 0.114, *p* < 0.01), and negatively correlated with green purchasing intention (*r* = –0.121, *p* < 0.01). ALVs are positively correlated with green purchasing intention (*r* = 0.351, *p* < 0.01), and EGVs are negatively correlated with green purchasing intention (*r* = –0.090, *p* < 0.01). The moderated variable has a low correlation with other variables.

**TABLE 2:** Descriptive statistics and correlation matrix (*N* = 977).

Variables	Gender	ALV	EGV	GPI	ME
Gender	—				
ALV	–0.093**	**(0.773)**			
EGV	0.114**	–0.016	**(0.721)**		
GPI	–0.121**	0.351**	–0.090**	**(0.830)**	
ME	0.008	0.181**	0.059	0.281**	**(0.808)**
Mean	0.549	6.480	3.636	5.778	4.349
SD	0.498	0.756	1.497	0.987	1.093

**p < 0.01 (two-tailed) and the diagonal is the arithmetic square root of the AVE value (bold values).

### Common Method Bias

All items in each questionnaire were completed by the same respondent, so a common method bias may exist (Podsakoff et al., 2003). Hence, Harman’s one-factor test is used to determine common method bias. An exploratory factor analysis was conducted for all items using SPSS 25, and the variance explanation rate for the primary factor before rotation is 29.628%, so there is no significant common method bias.

### Reliability and Validity Testing

The normal distribution of the sample data was tested through the skewness and kurtosis coefficients. The results show that the sampling data approximately conform to a normal distribution, indicating that a maximum likelihood estimation is possible. Cronbach’s alpha coefficient is used to test the internal consistency reliability of all the scales (as shown in Table 1). The results estimated by SPSS 25 show that the Cronbach’s alpha coefficient for each variable is greater than 0.7, which exceeds the recommended threshold of 0.7 (Hair et al., 2020).

The following procedures are recommended by Anderson and Gerbing (1988). A confirmatory factor analysis used AMOS 23, and the results show that the four-factor measurement model fits the sampling data well. The main goodness-of-fit indices are as follows: *χ^2^* = *222.636, df* = *71, χ^2^/df* = *3.136, RMSEA* = *0.047, CFI* = *0.976, GFI* = *0.968, TLI* = *0.969, IFI* = *0.976, NFI* = *0.966, SRMR* = *0.033*. As shown in Tables 1, 2, the standardized factor loadings for all latent variables range from 0.649 to 0.898 (*p* < 0.001), the AVE values for all constructs are greater than 0.5 (Hair et al., 2020), and composite reliability is greater than 0.7, so the criteria are fulfilled. This shows that there is good convergent validity for each variable. The arithmetic square root of the AVE value for each latent variable is greater than the correlation between this and other variables (Fornell and Larcker, 1981). A nested model comparison, which was proposed by Anderson and Gerbing (1988), was conducted to test discriminant validity, and the results are shown in Table 3. The chi-square difference between the restricted model and the default model is significant. The results of these analyses demonstrate that the empirical data for this study have sufficient discriminant validity.

**TABLE 3:** Discriminant validity test (*N* = 977).

Model comparison			Restricted model		Default model		Δ*df*	Δ*chi-square*	*p*
			*chi-square*	*df*	*chi-square*	*df*			
ALV	< – >	EGV	954.120	72	222.636	71	1	731.483	0.000
ME	< – >	ALV	1915.089	72	222.636	71	1	1692.453	0.000
ALV	< – >	GPI	1648.384	72	222.636	71	1	1425.747	0.000
ME	< – >	EGV	949.402	72	222.636	71	1	726.766	0.000
EGV	< – >	GPI	942.614	72	222.636	71	1	719.977	0.000
ME	< – >	GPI	1511.716	72	222.636	71	1	1289.079	0.000

### Hypothesis Testing

#### Test of Mediation Effect

This study uses Process v3.3 to test the multiple mediation effects and the subsequent moderated mediation model. The mediation effect test follows the procedure of Zhao et al. (2010); Hayes (2018) and uses the bootstrapping method in Process v3.3 with a resampling sample of 5,000, and the confidence level set to 95%. The results are shown in Table 4. The total mediation effect of ALVs and EGVs is significant, with an effect size of –0.0801 and the bootstrapping confidence interval (BootCI) value of (–0.1312, –0.0339), so H1 is supported. The mediation effect of ALVs is significant, with an effect size of –0.0628 and BootCI value of (–0.1108, –0.0200). The mediation effect of EGVs is significant, with an effect size of –0.0172 and BootCI value of (–0.0357, –0.0029), so H2 and H3 are supported. There is no significant difference in the two mediated paths, the Z value is –1.8313, and BootCI value is (–0.0968, 0.0010).

**TABLE 4:** Results for the mediation effect test (*N* = 977).

Hypothesis	Effect	Boot SE	95% Lower bound	95% Upper bound
Total effect	–0.2401	0.0630	–0.3638	–0.1164
Total indirect effect	–0.0801	0.0247	–0.1312	–0.0339
**Specific indirect effect**				
ALV	–0.0628	0.0233	–0.1108	–0.0200
EGV	–0.0172	0.0085	–0.0357	–0.0029

**FIGURE 3:**
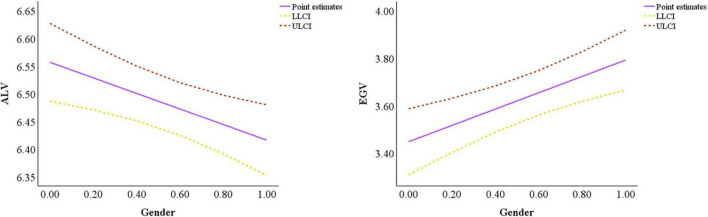
Comparison of the effect of gender on the dual values.

**FIGURE 4:**
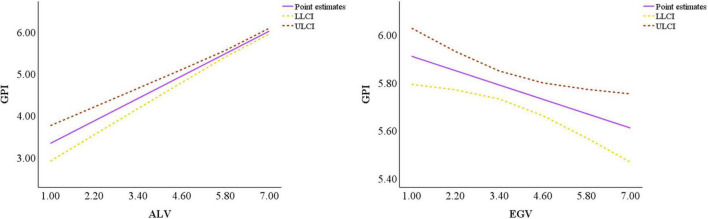
Comparison of the effect of dual values on green purchasing intention.

In the multiple mediation model, gender heterogeneity (using women as the reference group to compare to female’s and male’s dual values, the same below) negatively affects ALVs (B = –0.1406, β = –0.1859, *p* < 0.01) with BootCI value of (–0.2349, –0.0458) and positively affects EGVs (B = 0.3442, β = 0.2299, *p* < 0.001) with BootCI value of (0.1540, 0.5315). When independent variables are controlled, ALVs have a significant positive effect on green purchasing intention (B = 0.4469, β = 0.3424, *p* < 0.001), with a BootCI value of (0.3396, 0.5593), and EGVs negatively affect green purchasing intention (B = –0.0500, β = –0.0758, *p* < 0.05), with a BootCI value of (–0.0913, –0.0101). Figure 3 shows the difference in the paths for the effect of gender heterogeneity on the dual values, and Figure 4 shows the difference in the relationship between the dual values and green purchasing intention under controlling the influence of the independent variable.

#### Testing the Moderated Mediation Effect

This study refers to the test procedure of moderated mediation effect (conditional process analysis) that is proposed in the studies by Hayes (2018) and Hayes and Rockwood (2020). Setting the sample to 5,000 and the confidence level to 95%, model 59 is used to determine the moderated mediation effect (uses original data for gender). The statistical results are shown in Table 5. For mean of ME minus one SD, the mediation effect of ALVs is significant, with a BootCI value of (–0.1502, –0.012). For mean of ME plus one SD, the mediation effect of ALVs is not significant, with a BootCI value of (–0.0858, 0.0022). These results show that there is a significant moderated mediation effect (Hayes, 2018; Liu et al., 2018). Similarly, for mean of ME minus one SD, the mediation effect of EGVs is not significant, with a BootCI value of (–0.0368, 0.0076); for mean of ME plus one SD, the mediation effect of EGVs is significant, with a BootCI value of (–0.0646, –0.0051). These results show that the mediation effect of dual values is significantly moderated by ME (Liu et al., 2018).

**TABLE 5:** Results for moderated mediation effect (*N* = 977).

Mediator	Mean, mean ± 1SD	Effect	Boot SE	95% Lower bound	95% Upper bound
	3.2554	–0.0718	0.0363	–0.1502	–0.0120
ALV	4.3488	–0.0552	0.0195	–0.0960	–0.0199
	5.4422	–0.0401	0.0217	–0.0858	0.0022
	3.2554	–0.0108	0.0112	–0.0368	0.0076
EGV	4.3488	–0.0192	0.0089	–0.0388	–0.0046
	5.4422	–0.0298	0.0152	–0.0646	–0.0051

In terms of the direct path, for mean of ME minus one SD, gender heterogeneity significantly influences green purchasing intention, with a BootCI value of (–0.4763, –0.1524). For mean of ME plus one SD, the BootCI is (–0.1830, 0.1415). This result shows that gender heterogeneity does not have a significant effect on green purchasing intention, so the direct path is also significantly moderated by ME.

**FIGURE 5:**
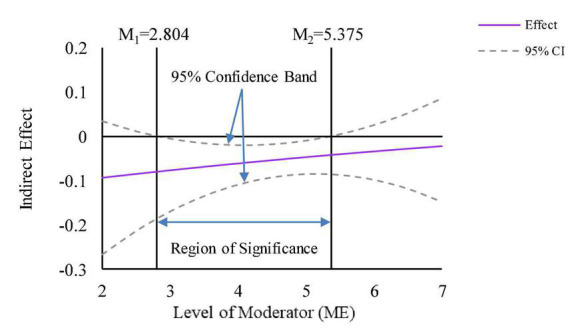
Moderated mediation effect of Media exposure (ME) (mediator = ALVs).

**FIGURE 6:**
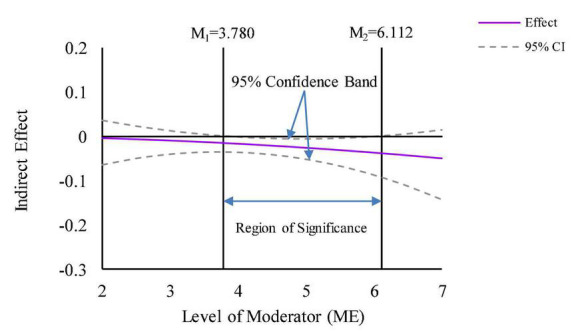
Moderated mediation effect of ME (mediator = EGVs).

This paper depicts the visualization following the Johnson-Neyman approach recommended by Hayes (2018) and yields specific values for the 95% confidence band and the region of significance. The plots for moderated mediation effects use the moderated effect for mean and mean of ME ± 1SD and corresponding confidence intervals. Figure 5 shows that ME negatively moderates the mediation effect of ALVs, compared to women. As ME increases, the negative mediation effect of male’s ALVs gradually decreases. For raw values of ME that range from 2.804 to 5.375 (Z-standardized values that range from –1.470 to 0.946), the indirect effect of gender heterogeneity on green purchasing intention through ALVs is significant. Figure 6 shows that ME has a weakly positive moderating effect on the mediation effect of EGVs, and the negative mediation effect of EGVs increases slightly as ME increases. For raw values for ME that range from 3.780 to 6.112 (Z-standardized values that range from –*0.455 to 1.618*), the indirect effect of gender heterogeneity on green purchasing intention through EGVs is significant. Therefore, H4 and H5 are supported.

**FIGURE 7:**
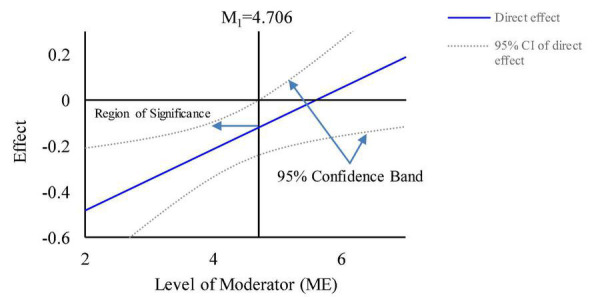
Moderated effect of ME on the direct path.

Similarly, Figure 7 shows that ME has a strongly negative moderating effect on the direct path, and the negative effect of gender heterogeneity on green purchasing intention decreases as ME increases and becomes positive at a later stage. Figure 7 also shows that for original values of ME of less than 4.706 (Z-standardized values are less than 0.327), the direct effect is significant.

## Discussion, Implications, and Limitations

### Discussion and Conclusion

This study determines the influencing mechanism for gender heterogeneity on green purchasing intention through ALVs and EGVs, empirically tests the mediation effects of the dual values, and compares the effect size and significance for two mediated paths. It also explores the moderating effect of a contextual variable on the mediation model.

First of all, in contrast to previous studies that use gender as a moderator, this study uses gender as a predictor variable and constructs a moderated mediation model. The results are in agreement with those of previous studies (e.g., Mostafa, 2007; Lee, 2009; Beynon et al., 2010) in showing that women care more for the environment than men and that gender differences in terms of green purchase are repeatable and verifiable across nation, time, and culture. This study successfully determines the specific path and achievement mechanism for the effect of gender heterogeneity on green purchasing intention that was initially posited in prior studies such as Fukukawa et al. (2007) and Mostafa (2007). This interesting finding broadly supports the work of other literature in this area that gender heterogeneity has an opposite effect on ALVs and EGVs and showing that men are more selfish than women (Mostafa, 2007; Pinto et al., 2014). This reproduces the male’s task-oriented style in the context of China’s green consumption. A possible explanation for this is that men are more concerned with non-emotional self-purpose (Meyers-Levy, 1988; Lan et al., 2009), so they are self-centered and have a weaker sense of social belonging (Pinto et al., 2014).

Furthermore, another results of this study support the hypothesis that gender heterogeneity significantly affects green purchasing intention by activating both ALVs and EGVs. Men exhibit a lower degree of altruism than women, so green purchasing intention is inhibited. Men are also more egoistic, which negatively affects green purchasing intention. This reveals the specific causes of males’ ineffective environmental behavior more clearly than previous studies. It is concluded that men have a greater attitude–behavior gap than women under the same conditions. This evidence further shows that separating the dual values significantly decreases green purchasing intention, so positive attitude is not translated into green purchasing behaviors.

Second, this study verifies the concept that social context factors are an important interference factor in the formation of green purchasing intention and the repetition of environmental information is partially effective, but has only a limited role. The results of this study demonstrate that ME significantly moderates the mediation model, so as ME increases, green purchasing intention due to male‘s ALVs increases slightly, but EGVs have a greater negative effect on green purchasing intention. These results corroborate the conclusions of Yang et al. (2020), who noted that high-frequency dissemination of environmental crisis information by the media increases an individual’s perception of the significance of environmental problems (Yang and Zhang, 2021) and activates perceived environmental responsibility (Mi et al., 2020), which increases concern for the environment (Kiatkawsin and Han, 2017; Costa et al., 2021).

Contrary to expectations, this study shows that ME does not weaken the negative mediation effect of EGVs. This may be explained by the conclusions of Lee (2009) and Ivanova et al. (2019), who stated that the perceived seriousness of environmental problems due to media reports and the ecological perceptions of whole society assign symbolic value to green purchasing activities. Men construct social status and self-image by purchasing green products, so this activity reinforces male’s self-enhancement values (Pinto et al., 2014). Taken together, these results show that green purchasing activities, which reflects the characteristics of public-sphere environmental behaviors, are more easily embodied on a personal moral level, due to selflessness and man–nature orientation. Obviously, these findings shed new light on the different rules for the evolution of the mediation effects of ALVs and EGVs under the interference of moderator in detail. Meanwhile, the negative moderating effect on the direct path reconfirms this inference.

Third, this study increases understanding of the size of the attitude–behavior gap, which depends on the trade-off between altruistic appeals and egoistic appeals, gender differences, and moderated role of social contextual factors. The joint influence of ALVs and EGVs on green purchasing intention is significant. This finding further supports that the results of previous studies use consider dual appeals to establish a long-term mechanism for green consumption (Yang et al., 2020) and corroborates the findings of Le et al. (2019), Szabo and Webster (2021) who concluded that separating dual appeals may create much token environmental behavior. Thus, individual’s egoistic appeals are an essential element in reducing the negative impact of EGVs on green purchasing intention. Hereby, a widespread attitude–behavior gap indicates that the current green market does not yet balance consumers’ egoistic appeals and altruistic appeals, misleading and ambiguous information still pervades the current green market, so green initiatives have a low perceived value (Woo and Kim, 2019; Luo et al., 2020), and individual rationality cannot be balanced with collective rationality. The logical origin of green purchasing is still dominated by collective rationality, thus weakening the influence of values on green purchasing intention, so the attitude–behavior gap is broadened.

Finally, this study is one of the first attempt to determine gender differences in the attitude–behavior gap: ceteris paribus, men exhibit a greater attitude–behavior gap. These differences are partly explained by Mostafa (2007), Lee (2009), Yang and Zhang (2021), who noted that women exhibit a higher sense of social responsibility, environmental concern, and ecological consciousness than men. Furthermore, media is a potential moderator of the size of the attitude–behavior gap. ME plays a positive role in narrowing the gap by actively promoting ALVs. However, excessive promotion of EGVs results in extravagant egotism attribution bias in men, so the diversity and dynamics of contextual factors mean that reducing the attitude–behavior gap is a complex and lengthy process. These preliminary results show that many attitude–behavior gaps are formed at the attitude–intention stage.

### Implications

#### Theoretical Implications

This study contributes to existing knowledge of gender differences in terms of green consumption using ALVs and EGVs from the VBN theory, determining the different effects of gender heterogeneity on the dual values for assumptions of bounded morality and bounded self-interest, and determines the process by which these values are created. The validity of these results is also tested from crosscountry and crosscultural perspectives to construct a solid theoretical basis for the subsequent establishment of ecological values and an ecological society.

Furthermore, this study determines the different influencing mechanisms of gender heterogeneity on green purchasing intention through ALVs and EGVs, clarifies the differences in generation logic for green purchasing intention with respect to these dual values, and theoretically determines the origin and dynamic mechanism for green purchasing intention. Meanwhile, the endogenous force of the development of this intention is identified and systematically determines formation rules for green purchasing intention, which gives a theoretical reference for establishing a long-term mechanism to promote green consumption. This study gives a new understanding of the attitude–behavior gap from the perspective of the causal chain involving attitude, intention, and behavior and demonstrates the complex reasons for an attitude–behavior gap by decomposing ALVs and EGVs. This allows a new theoretical framework for detecting the gap.

Moreover, this study also verifies the validity and limitations of media repetition strategies in the context of green consumption and determines the generation logic and the optimal path for positive and negative effects of ME. This provides a theoretical basis for optimizing media persuasion strategies, scientifically implementing group segmentation and achieving accurate persuasion, which further expands the theoretical research perspective for this field.

#### Practical Implications

Environmental practitioners must verify the causal path for the generation and development of green purchasing intention or behavior, and abandon fuzzy decision-making in terms of traditional green claims to account for the different effects of gender heterogeneity on the dual values and green purchasing intention. It is also necessary to determine the key elements that contribute to green purchasing intention. Practitioners and policymakers must design and disseminate persuasive information, formulate an award policy, and account for intervention from consumer decision-making to design strategies that pertain to the characteristics of information reception and the processing of individuals of different genders. To activate male’s ALVs and allow these to assume a guiding role, green consumption should be assigned symbolic value such as success, capability, and ambition (i.e., personal success, demonstration of capabilities or status).

If green consumption depends on government initiatives in some regions, the effect of the government model is important, so governments must assume the role of a green policymaker and manifest the role of green life practitioner, thus accelerating the creation of symbolic values that are embedded in green consumption and associate this activity with representative functions such as ability, ambition, success, and social status. In terms of women, green practitioners must attach more importance to self-transcendent values and activate female’s environmental emotions and humane concerns to create concern about the scarcity of environmental resources for future generations (Pinto et al., 2014).

In addition, media plays the roles of participant, actor, guide, and supervisor in the construction of ecological civilization, but there is a discrepancy between environmental advocates expecting great things of the media and the weak moderated mediation effects, so the media needs to self-reflect on the shortcomings of current persuasion strategies. Environmental practitioners might consider optimizing the selection of a media platform and initiating an information diffusion strategy to establish media credibility, create trust in the media, and ensure self-discipline with respect to non-environmental behavior. The different moderating effects of media heterogeneity and media strategy heterogeneity on the generation of green purchasing activities based on precise audience segmentation are also worthy of study. A two-sided information strategy and a one-sided information strategy could be developed to increase the fluency of individual information processing and could induce active thought, rather than passive preaching. This would achieve optimal promotion of the audience’s positive emotion toward green consumption. In particular, segmenting the audience in terms of differences in information processing and differences in the values of female and male subgroups could be used to continuously improve green product attributes and media communication strategies to match gender characteristics (female vs. male subgroups).

Moreover, to minimize the attitude–behavior gap, environmental practitioners could apply global and dynamic methods to diagnose the complex causes of this gap and to prevent the evolution of green product promotion into moral kidnapping. Any repair strategy for this gap must be dynamically adjusted in terms of changes in gender characteristics, economic conditions, and social contexts, to ensure precise repair, grouping repair, and subregion repair and reduce negative interference from uncertainty factors during restoration. For instance, the media could account for female’s caring feelings to construct a positive picture of man and nature in harmonious coexistence and highlight the win-win outcome of environmentally friendly behavior for environment, family, and friends.

### Limitations and Future Study

This is a cross-sectional study, so the causal relationships between variables inferred. These must be verified using longitudinal sampling data. Meanwhile, the sample includes only urban residents so potential differences in green consumption caused by the urban–rural duality that exists in China are not measured. Future studies should use samples from rural residents or obtain samples from both urban and rural residents, to increase the universality of these findings, and further determine the difference in the performance of the green purchasing activities of urban and rural residents.

## Data Availability Statement

The raw data supporting the conclusions of this article will be made available by the authors, without undue reservation.

## Author Contributions

XY and JJ carried out the conceptualization. XY and S-CC involved in the methodology. XY conducted the investigation. XY and JJ performed the formal analysis. S-CC supervised the study. SK performed the visualization. SK, TN, and JJ validated the manuscript. XY, SK, TN, JJ, and S-CC involved in writing the original draft preparation and performed the writing, reviewing, and editing. All authors have read and agreed to the published version of the manuscript.

## Conflict of Interest

The authors declare that the research was conducted in the absence of any commercial or financial relationships that could be construed as a potential conflict of interest.

## Publisher’s Note

All claims expressed in this article are solely those of the authors and do not necessarily represent those of their affiliated organizations, or those of the publisher, the editors and the reviewers. Any product that may be evaluated in this article, or claim that may be made by its manufacturer, is not guaranteed or endorsed by the publisher.
